# An Innovative Technique Using a Stainless Steel Double Die Pin Retained Cheek Plumper in Complete Denture Esthetics: A Case Report

**DOI:** 10.7759/cureus.6197

**Published:** 2019-11-19

**Authors:** Sriharsha Pudi, Shanthipriya Kota, Karthik K V G Ch, Sainath Reddy Kaladi, Rajasekhar Reddy Gade

**Affiliations:** 1 Prosthodontics and Crown & Bridges, MNR Dental College and Hospital, Hyderabad, IND; 2 Prosthodontics, MNR Dental College and Hospital, Hyderabad, IND; 3 Prosthodontics, Army College of Dental Sciences, Hyderabad, IND; 4 Conservative Dentistry and Endodontics, MNR Dental College and Hospital, Hyderabad, IND; 5 Prosthodontics and Crown & Bridges, St. Joseph Dental College and Hospital, Eluru, IND

**Keywords:** unconventional dentures, sunken cheeks, cheek plumper

## Abstract

In present times individuals are more concerned about esthetics. Aging leads to a high impact on external facial esthetics resulting in slumped cheek leading to undesirable facial esthetics. Cheek plumper is a commonly used prosthesis to enhance the support of sunken cheeks providing better esthetics. Die pins retention for sunken cheek patients is advantageous due to its precise fit, excellent esthetics, and stability during various functional movements. This innovative approach helps in accomplishing prosperity of the patient. This case report describes about a simple, effective, and noninvasive treatment strategy to re-establish the sunken cheeks utilizing cheek plumper which is appended to the conventional complete denture, using stainless steel double die pins.

## Introduction

A prosthodontist assumes an imperative role in prosthodontic rehabilitation which does not intend to simply replace the missing teeth, but rather additionally re-establish the facial support [1]. Cheeks are a vital part of facial esthetics due to their extreme visibility. The support provided by the teeth, the ridges or the dentures determine the form of the cheeks [2]. Aging leads to a high impact on external facial esthetics due to early tooth loss, alveolar resorption, and reduced tonicity of musculature. This results in slumped or hollow cheek leading to undesirable facial esthetics [3]. Cheek plumpers are basically to support and plump the cheek to give a youthful appearance. It is particularly helpful for patients who have lost their teeth and part of the maxillary bone as a result of a traumatic injury. Undetectable cheek plumper has some disadvantages like increased weight, difficulty in insertion, muscle fatigue; interference with masseter and buccinator muscle function and coronoid process of the mandible [2]. It can also not be used in patients with limited mouth opening. Some authors have used magnets [1, 4-5] and push button detachable cheek plumper [3, 6-7] as attachments to overcome the demerits of undetectable cheek plumper. But these magnets and push buttons as attachments also have some disadvantages. Hence, this clinical report focuses on how to enhance facial esthetics of completely edentulous patients with sunken cheeks with the help of stainless steel double die pins as a detachable cheek plumper.

## Case presentation

This clinical case report was that of a stainless double die pin retained cheek plumper in complete denture esthetics treated at JSS Dental College and Hospital, a constituent college of Jagadguru Sri Shivarathreeswara University, Mysore, Karnataka. 

**Figure 1:**
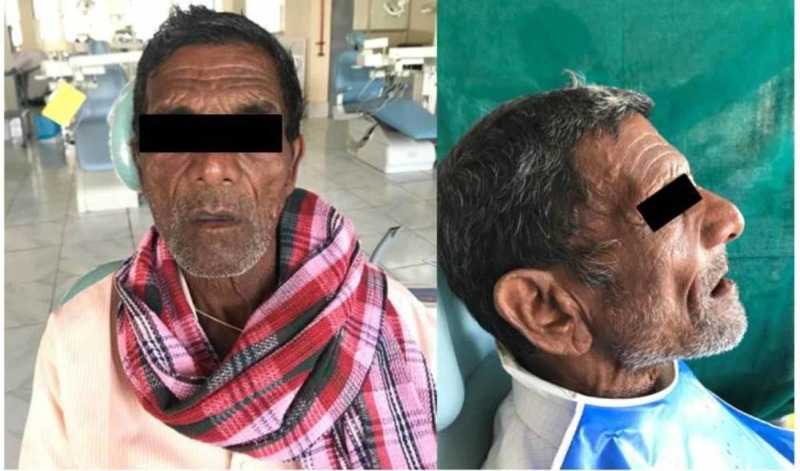
Preoperative photograph of the patient.

A 75-year-old male patient reported to the Department of Prosthodontics and Crown & Bridge with the chief complaint of missing teeth and sought replacement due to unesthetic appearance (Figure 1). He gave a history of teeth extracted over a period of two years due to periodontal problem and decay. He was edentulous since three months. On examination one of the significant findings was poor esthetics, unsupported oral musculature, and slumped cheeks. The patient required complete dentures with some form of cheek support. Based on the patient’s needs a treatment plan was formulated. It was chosen to give the patient upper and lower complete dentures with detachable cheek plumper for the maxillary denture.

Clinical procedures

**Figure 2:**
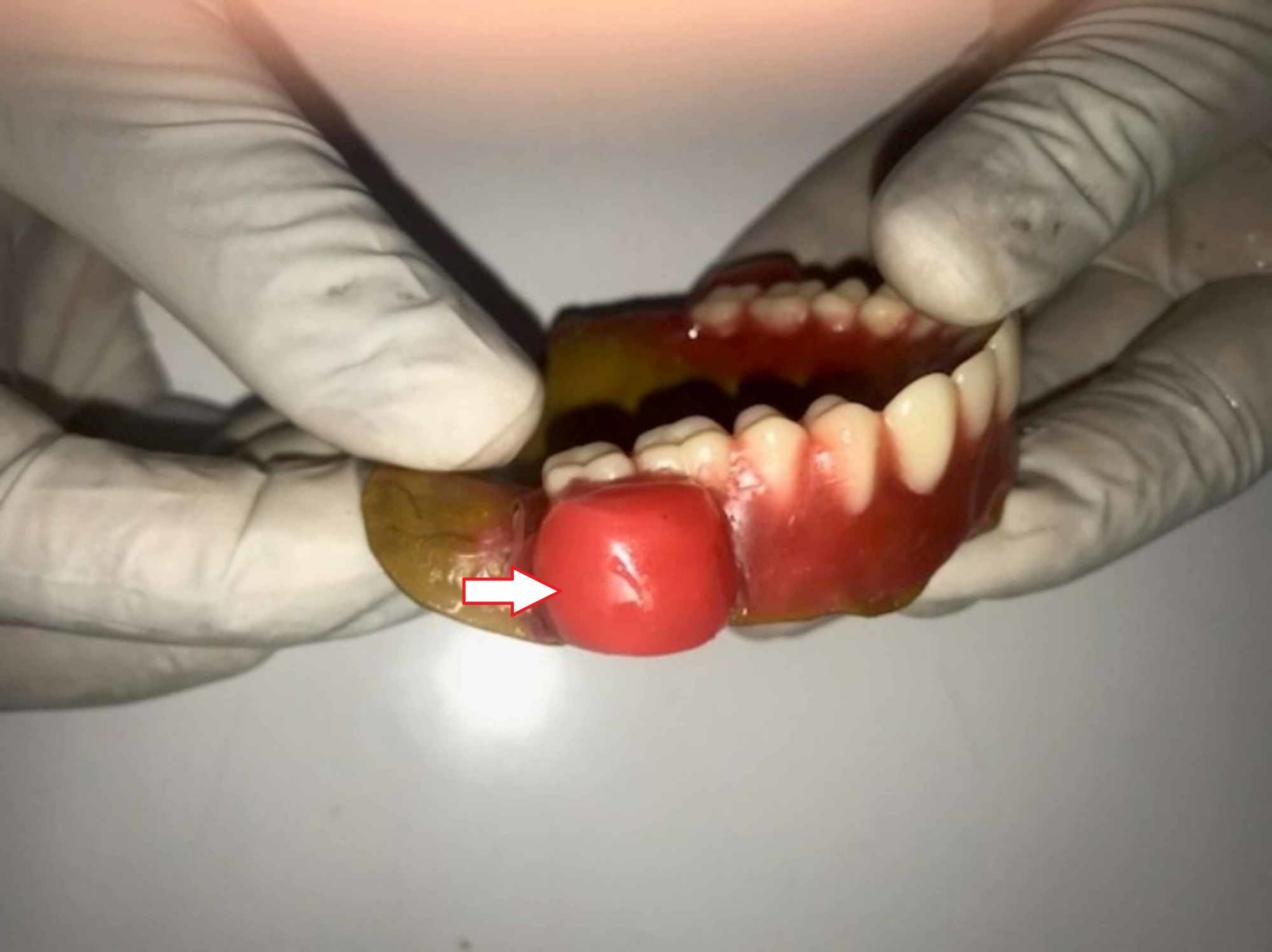
Try-in stage cheek plumper was made using wax.

Maxillary and mandibular impressions were made using impression compound (DPI PINNACLE, Dental products of India, Mumbai) and dental plaster was poured to obtain the primary cast. Spacers were adapted over the cast and the special trays were fabricated using autopolymerizing acrylic resin. Border molding was done using low fusing impression compound (DPI PINNACLE tracing sticks, Mumbai) and final impressions were made with zinc oxide eugenol impression paste (DPI, Mumbai). Dental stone was poured over the impression to obtain the master cast. Jaw relations were recorded using modelling wax in conventional method and casts were mounted on to the three-point articulator. Teeth arrangement was done using premadent teeth and a wax set-up was tried in the mouth to check esthetics, phonetics, occlusal vertical dimension, and occlusion. At the try-in stage, cheek plumper was made using wax (Figure 2) as separate portions on the buccal surface of the complete trialdenture. They were superficially appended to the buccal surfaces on the right and left side and tried in the mouth to determine the amount of desired cheek support appropriate for comfort, function, and esthetics. Trial dentures were coated with a thin layer of petroleum jelly to facilitate the easy removal of the plumpers for processing. These plumpers were located between the first molar and second molar region of the maxillary denture flange. The adjustment in the appearance with and without wax-up cheek plumper was apparent and was readily accepted. They were designed according to the intraoral space availability which was necessary to enhance the appearance and thickness which otherwise would interfere with functional movement.

Laboratory procedures

**Figure 3:**
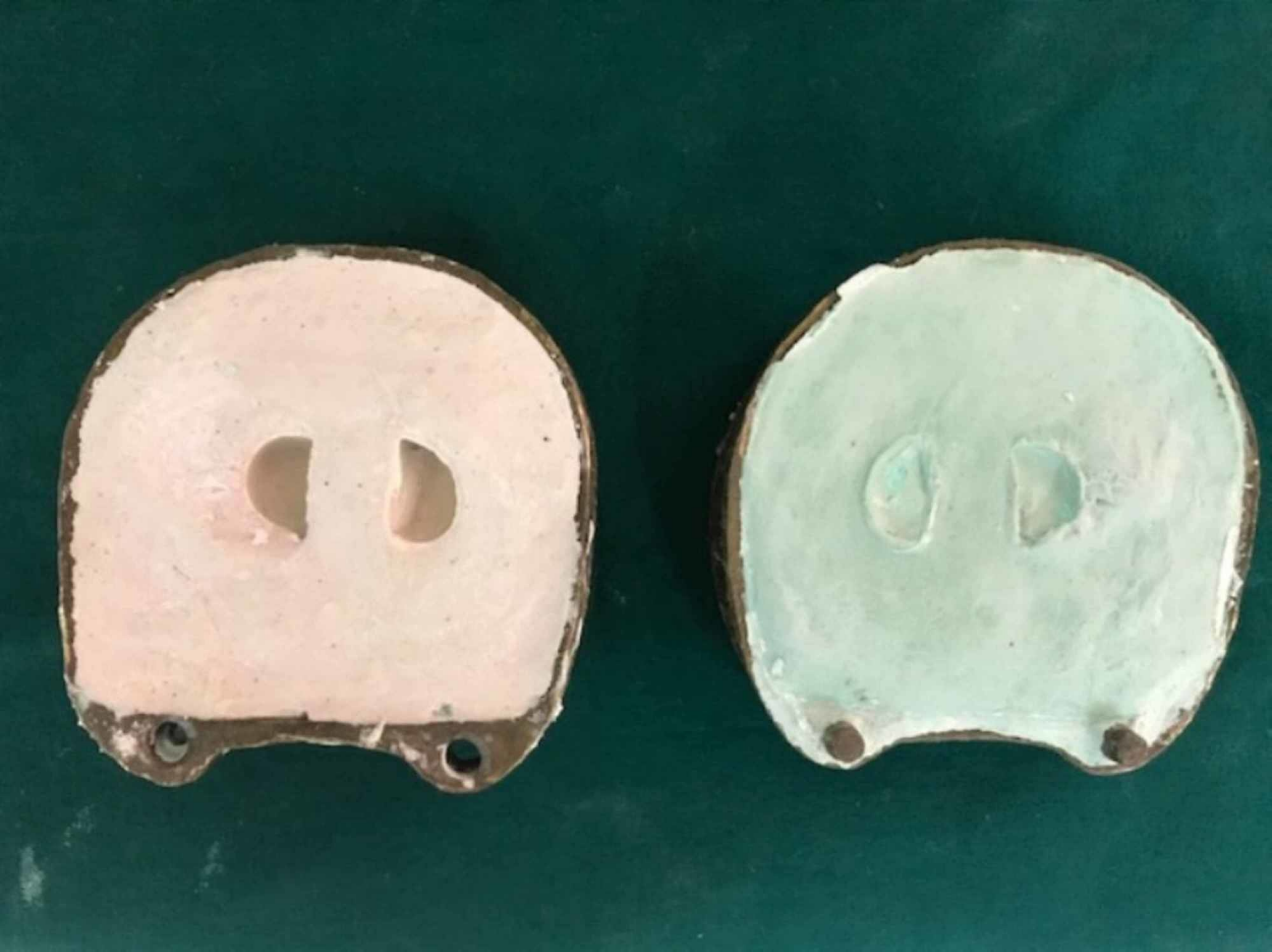
Resultant mold space after dewaxing.

**Figure 4:**
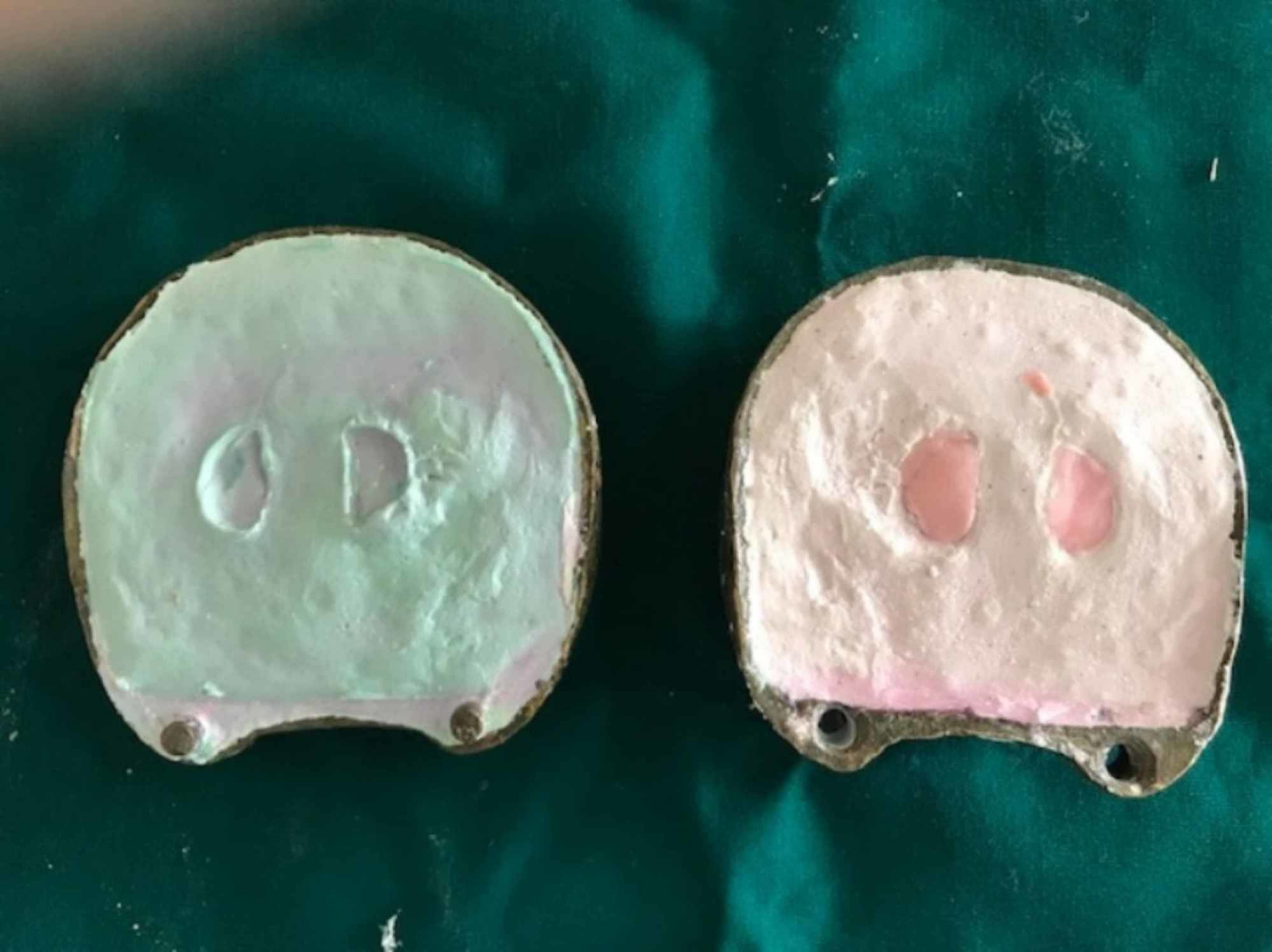
Cheek plumper packing.

**Figure 5:**
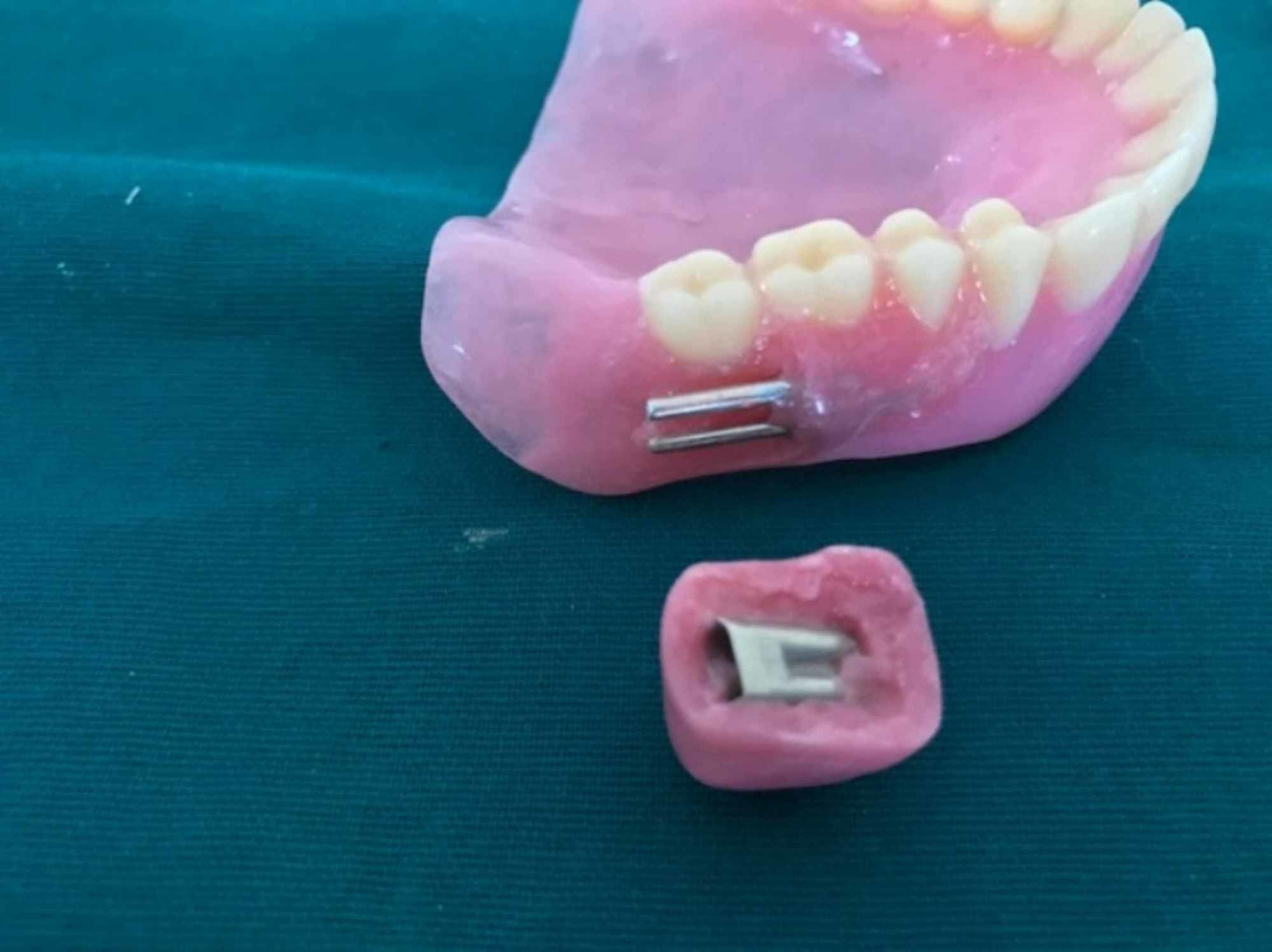
Die pins were placed in a denture base and sleeves were placed in a plumper.

**Figure 6:**
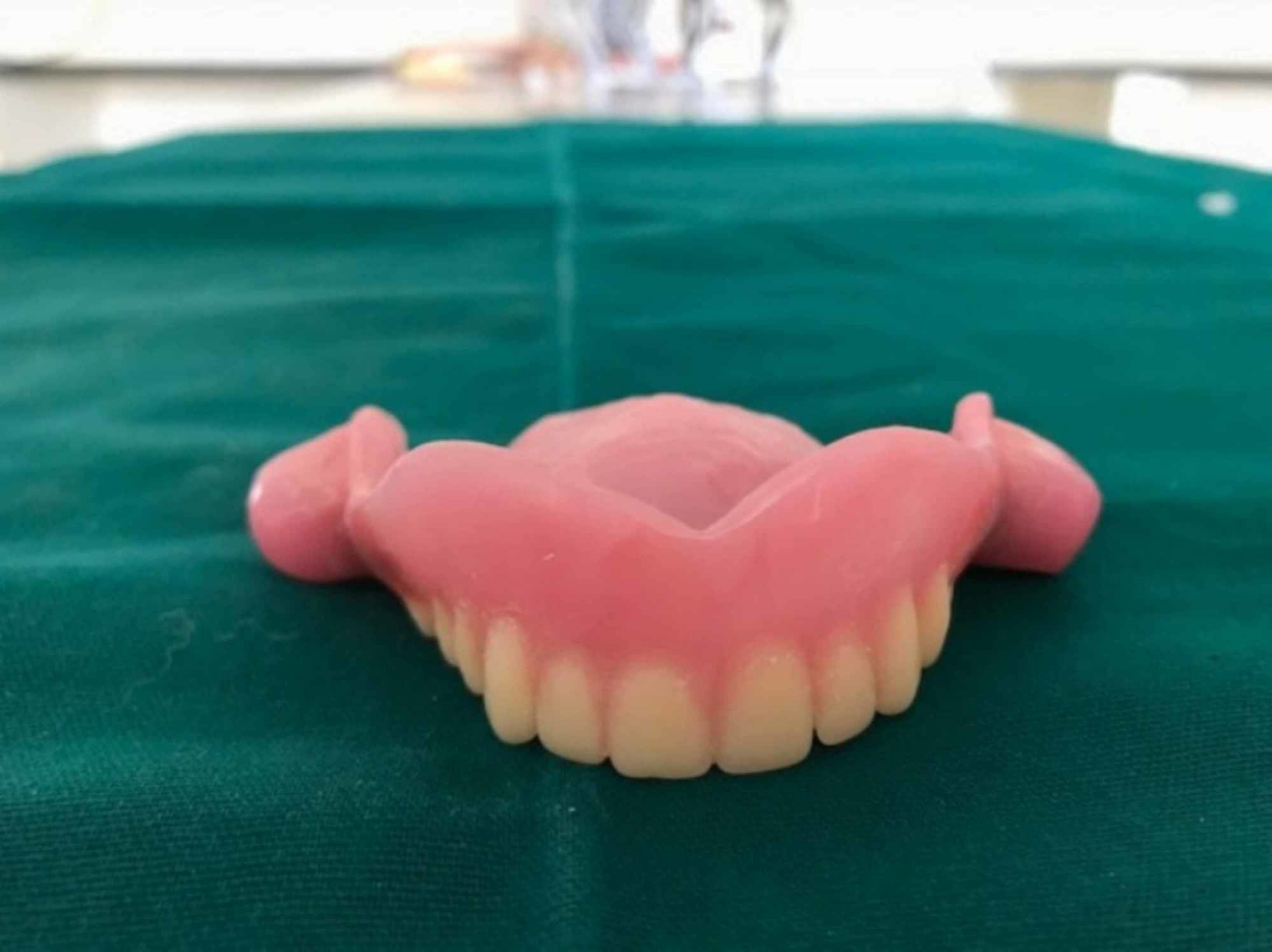
Plumpers attached to dentures.

**Figure 7:**
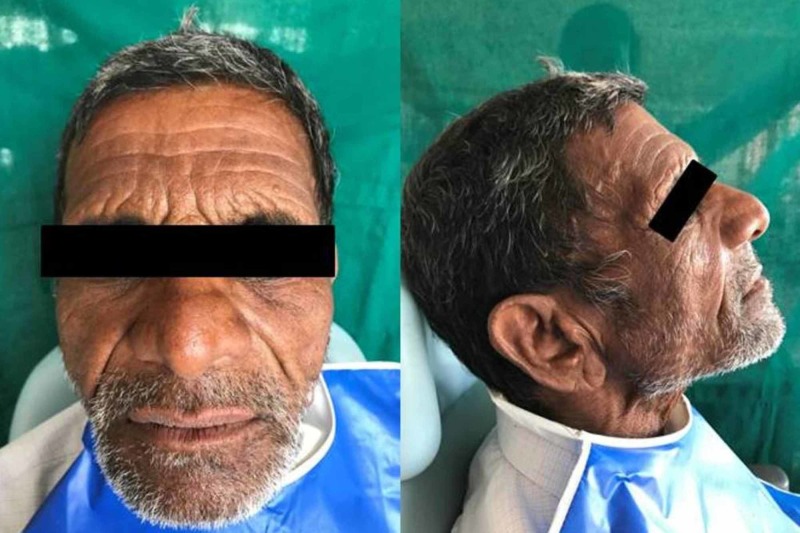
Postoperative photograph of the patient.

The plumpers and dentures were separately acrylized in the conventional way. Denture flasking and dewaxing procedures were finished separately for the final denture and cheek plumpers. The resultant mold space (Figure 3) was then packed with heat polymerizing acrylic material (Figure 4) and curing procedure was completed. After curing, the cured final prosthesis and plumpers were retrieved. Trimming, finishing, and polishing procedures were performed. After finishing and polishing, slots were made in the buccal surface of the denture for the die pins and the attachment surfaces of the plumpers also trimmed to make space for sleeves. Double die pins were placed in the denture base and the sleeves were placed on the cheek plumper (Figure 5). The angulations and location of the die pins and their sleeves were evaluated so that there be no discrepancy and the double die pins could easily be seated in their respective sleeves. After conforming the location double die pins were sealed with autopolymerizing resin on the denture base and sleeves on the cheek plumpers. Holes of the sleeves were blocked with wax to prevent the flow of the acrylic resin into the sleeves. Autopolymerizing resin was allowed to set properly. The sleeves were inserted into the die pins and then little acrylic resin in dough stage was carefully kept on the shaft of the pins on buccal surface of the denture base. The resin was not allowed to flow beyond the shaft of the die pins for proper insertion and removal of the sleeves from the double die pins. After the resin had set, the plumper could easily be removed. Trimming and polishing of the irregular surfaces were done and the prosthesis was delivered (Figures 6-7).

The patient was educated on the use and positioning of the plumpers and dentures were delivered after evaluating them for fit and esthetics. On the recall of 48 h the patient did not possess any problems with speech or mastication.

## Discussion

Denture esthetics is not limited to selection of teeth based on factors like form, shape, color, and arrangement of the teeth [4]. With increasing age, treatment modalities become even more challenging because of an extreme change in tissue atrophy; folds and creases of face occurs. Loss of posterior teeth results in loss of cheek support, which tends to move medially to meet laterally encroaching tongue. There is even change in cheek contour due to loss of the vertical dimension of occlusion. Factors such as loss of subcutaneous fat, buccal pad of fat, and elasticity of the connective tissue are the reasons of the sunken cheeks that are seen in the aged persons [3, 8]. Corrections of slumping of cheeks can be accomplished by various methods like reconstructive plastic surgery, injecting the botulinum toxin (BOTOX) in the facial muscles [9], and different types of prostheses. The plastic surgery is a traumatic procedure which leaves behind the postsurgical scar, sometimes contraindicated in old patients suffering from systemic diseases. Although these modalities may be effective, they have a variety of disadvantages among them, including cost, time to onset, skin irritation, and allergic skin reactions [4]. Cheek plumpers or cheek lifting appliances have been used previously for the purpose of improving esthetics and psychological profile in patients. Single piece prosthesis may cause inconvenience for the patient. To overcome the disadvantages of conventional cheek plumper, detachable cheek plumpers are now in use that have advantages of reduction in height, easy insertion and removal, prevention of muscle fatigue, cleaning and allowing patient to wear only denture without cheek plumper. Detachable cheek plumpers such as magnets, soft liners, wires, and customized attachments have been used but they all have disadvantages that prevent their regular uses. The attachments should be cost effective, resist rotation or movement of plumpers while in function, and long acting. Magnetic retention for slumped cheek patients is beneficial due to good retentive forces, small size, and automatic re-seating; however, due to harmful effects of magnetic field on the health of the oral tissues and loss of magnetic property over a period of time it requires frequent replacement [1, 4-5]. Press button retained cheek plumpers are now most commonly used that have the advantages of small and light weight, snug fit, and cheap; on the other hand due to poor corrosion resistance and food lodgement it was not advised as a retained cheek plumper [6-7, 10]. Recently double die pins were used to retain the cheek plumpers [7]. Double die pins were placed on the plumpers and the sleeves for the pins were placed in the denture base. In this case double die pins were placed in the denture base and sleeves for the pins were placed on the plumpers due to smaller size of cheek plumpers and also stainless steel double die pins were used to resist corrosion. This clinical report focuses on how to enhance facial esthetics of completely edentulous patients with sunken cheeks with the help of stainless steel double die pins as a detachable cheek plumper. The die pin retained cheek plumper prosthesis successfully restored the contour of the cheek, improved the esthetics and psychological well-being of the patient. Double die pin retained detachable cheek plumper simplifies the procedure and helps the patient to attach or detach the cheek plumpers as per their convenience.

Advantages of die pin retained cheek plumpers:

1. Precise fit

2. No rotation or movement while function during insertion/removal of prosthesis

3. Good strength

4. Corrosion not reported

5. Economical

6. Double die pins can be used for either small or big size cheek plumpers

7. Can be used in patients with compromised dexterity.

## Conclusions

A new prosthetic treatment with stainless double die pin retained cheek plumper in complete denture which was simple, effective, and noninvasive treatment acts as an alternative to improve facial appearance in a patient with sunken cheeks and also boost the self-esteem of the patient by improving his appearance. The use of double die pins in the present case report demonstrates a fundamental change in approach from the conventional methods. Die pins retention for sunken cheek patients is advantageous due to its precise fit and also provides excellent esthetics and stability during various functional movements. This innovative approach helps in accomplishing overall prosperity of the patient.
